# Strength Assessment of Water–Glass Sand Mixtures

**DOI:** 10.3390/gels9110850

**Published:** 2023-10-27

**Authors:** Toshiyuki Motohashi, Shigeo Sasahara, Shinya Inazumi

**Affiliations:** 1 Osaka Bousui Construction Co., Ltd., Osaka 543-0016, Japan; motohashi@obcc.co.jp; 2 Fuji Chemical Co., Ltd., Osaka 534-0024, Japan; s-sasahara@fuji-chemical.jp; 3 Shibaura Institute of Technology, College of Engineering, Tokyo 135-8548, Japan

**Keywords:** chemical injection, consolidation drainage triaxial compression test, hydrogel, sand-gel, small angle X-ray scattering test

## Abstract

For years, the chemical injection process has aided construction works by increasing the strength and water-sealing efficiency of sandy soil. Despite its growing popularity in projects, such as seismic strengthening and liquefaction mitigation, a unified understanding of how chemically treated soil develops its strength, especially under static conditions, remains elusive. Some studies have proposed that strength is derived from the tensile effects of dilatancy, where shearing of the sandy soil causes expansion, creating tension in the interstitial hydrogel and resulting in negative pressure that consolidates the soil particles. Other studies, however, attribute this strength development to the volumetric shrinkage of the hydrogel, which the authors argue confines and compresses the sandy soil particles. Challenges are encountered with this theory, particularly with respect to the consistency of the volumetric shrinkage measurements and the timing of these measurements in relation to changes in soil strength. The aim of the current research is to shed light on this mechanism by using consolidation drainage triaxial compression (CD) tests to measure the cohesive strength and internal friction angle of chemically enhanced soil. By eliminating the dilatancy-induced negative pressure effects and coupling this with an analysis of the molecular structure of the hydrogel, the present study provides an in-depth look at the strength development mechanism and its durability. This holistic approach not only fills in the existing gaps in the understanding of this mechanism, but also paves the way for optimized construction techniques.

## 1. Introduction

For many years, the chemical injection method has been widely used in construction projects as a supplementary method to increase the strength of sandy soil and improve the water-sealing efficiency. In recent years, due to its simplicity and flexibility, the method has also been widely used as the main part of construction projects, such as seismic reinforcement and liquefaction countermeasures, to protect existing infrastructures. For this reason, many studies have been conducted on the durability and dynamic behavior of bodies improved via chemical injection [1,2,3,4,5,6]. However, there is no unified view on the mechanism of the strength development of these bodies improved via chemical injection, even for static behavior; thus, the mechanism of strength development has yet to be clarified.

Mori and Tamura [7,8] and Mori et al. [9,10,11] focused on the tensile strength and negative pressure associated with dilatancy as the mechanism of the increase in strength brought about via chemical injection. When sandy soil improved via chemical injection is sheared, the sandy soil particles become unentangled and undergo volumetric expansion, at which time the hydrogel in the interstitial space among the sandy soil particles is subjected to tension. In addition, vacuum-based negative pressure is generated at this time, and the negative pressure pushes the sandy soil particles together. Mori and Tamura [7,8] and Mori et al. [9,10,11] further investigated the effect of negative pressure and discussed the strength development of sandy soil improved via chemical injection that is not affected by negative pressure.

Sasaki et al. [12] and Uemura et al. [13] focused on the volumetric shrinkage of hydrogel and proposed that the strength development of sandy soil improved via chemical injection can be explained by “confining pressure”, an effect of this volumetric shrinkage that restrains the sandy soil particles. According to their models, it is assumed that the loss in strength of the sandy soil improved via chemical injection can also be explained by volumetric shrinkage. However, this argument has the following problems. (1) The volumetric shrinkage of hydrogel cured using a chemical solution varies greatly depending on the measurement method, measurement size, and measurement environment. (2) All of the measurement data that have been published thus far are for hydrogel with a large volume (i.e., meth flask or specimen mold size), and it is not clear whether volumetric shrinkage occurs in the sandy soil particle interstices. (3) There is not a good time series correspondence between volumetric shrinkage and the increase or decrease in the strength of the sandy soil improved via chemical injection. Kaga and Mori [14] pointed out that the relationship between the strength of the improved sandy soil and volumetric shrinkage may be very slight.

Recently, the mechanism of strength development for sandy soil improved via chemical injection has been discussed in terms of “unconfined compressive strength”, which is mechanically ambiguous. Although unconfined compressive strength is an index value for construction control, Mohr–Coulomb’s failure criterion is applied to the strength of the ground, and the strength of the sandy soil improved via chemical injection should be discussed based on this fracture criterion.

In the present study, the mechanism of the strength development and the durability of the sandy soil improved via chemical injection are discussed based on the results of consolidation drainage triaxial compression tests (CD tests). The authors also analyze the change in the chemical structure of the solidified chemical solution itself (hereafter referred to as “hydrogel”) to gain insight into the mechanism of the strength development and durability. Specifically, CD tests are used to evaluate the cohesive strength and internal friction angle, which are the strength components of the sandy soil improved via chemical injection. These CD tests are used to eliminate the effect of negative pressure due to dilatancy. In addition, an analysis of the microscopic molecular structure of the hydrogel is used to comprehensively examine the mechanism and durability of the strength development of the improved sandy soil.

In the vast landscape of studies focusing on the strength development of sandy soil enhanced via chemical injection, there persists a lack of consensus on the governing mechanism, particularly within the context of static behavior. The present study represents a groundbreaking approach to understanding this mechanism by uniquely integrating CD tests with a microscopic molecular analysis of the hydrogel’s structure. Specifically, the authors employ CD tests to assess the cohesive strength and internal friction angle of the improved sandy soil, with the aim of removing the impact of negative pressure due to dilatancy—a pioneering step beyond previous methods. Taking it a step further, the analysis also delves into the microscopic molecular structure of the hydrogel, providing unparalleled, comprehensive insight into both the mechanism and durability of the strength development in chemically improved sandy soil. This integrated methodology offers a more holistic and precise perspective, addressing longstanding gaps and ambiguities in the field, and lays the foundation for more informed and effective construction practices in the future. 

## 2. Materials and Methods

### 2.1. Materials and Preparation of Specimens

**Figure 1:**
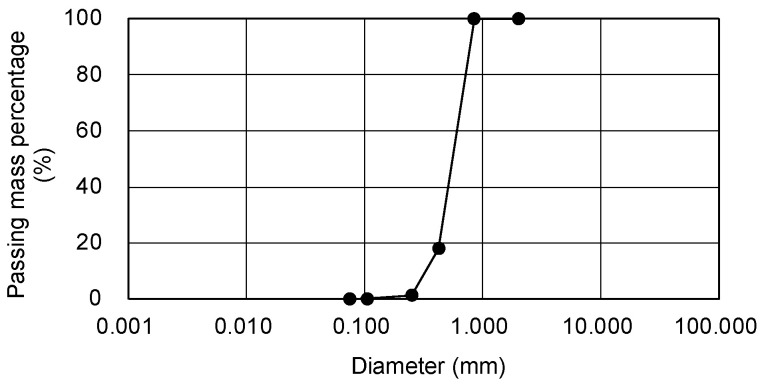
Particle size distribution curve of Tohoku silica sand No. 5.

Tohoku silica sand No. 5 was used for the sandy soil sample. As it does not contain fine-grained particles, it was judged to be a suitable material for evaluating the strength development of the sandy soil improved via chemical injection. The density of the sandy soil particles was ρ_s_ = 2.639 g/cm^3^ and the particle size distribution was as shown in Figure 1.

The chemical solution used in the tests was an acidic solution to which water glass was added; it solidifies in the non-alkaline range. This chemical solution is conventionally regarded as lacking stability for long-term strength due to the large volumetric shrinkage of the hydrogel. Three silica concentrations were used in the chemical solution: 7, 9, and 12%. Each silica concentration was calculated by dividing the weight of the silica content (SiO_2_) in the chemical solution by the total volume of the chemical solution.

Sand-gel and hydrogel specimens were prepared by solidifying Tohoku silica sand No. 5 with the chemical solution, while hydrogel specimens were also prepared by solidifying only with the chemical solution. The sand-gel specimens were prepared in 5 cm × 13 cm molds by the drop-in-water method. The gelation time was fixed at 30 to 60 min by adjusting the pH. The hydrogel specimens were also prepared in 5 cm × 13 cm molds. All the specimens were unmolded 60 min after gelation, shaped into 5 cm × 10 cm specimens, and cured in water.

Hydrogel specimens were also prepared in glass capillaries. Two types of glass capillaries were used: ϕ0.15 mm to simulate the pore spaces between the sandy soil particles and ϕ2 mm for the specimen used in the small-angle X-ray scattering tests (SAXS tests) described below.

The tests conducted in this study included the mechanical testing of sand-gel and hydrogel specimens, volumetric shrinkage testing of hydrogel specimens, and structural analysis of hydrogel specimens.

### 2.2. Mechanical Tests

The mechanical tests carried out on the sand-gel specimens were unconfined compression tests (UC tests), unconfined compression tests under water immersion (UC tests under water immersion), and consolidation drainage triaxial compression tests (CD tests). The UC tests were performed to investigate the variability of the sand-gel specimens. The silica concentration of the chemical solution was 12%, the material age of the specimens was 7 days, and the number of UC-tested sand-gel specimens was 24. The UC test under water immersion is a compression test without a rubber sleeve and without the application of confining pressure in the cell used in the triaxial compression tests. The UC tests under water immersion conducted in this study were performed using a simple method in which the sand-gel specimens were compressed in a plastic bag filled with water [15]. UC tests under water immersion can eliminate the negative pore pressure that has been generated inside the sand-gel specimens, which affects the strength development of the specimens. The CD tests were also conducted to eliminate the effect of the negative pore pressure generated inside the sand-gel specimens. Back pressure of 49 kN/m^2^ was applied to ensure saturation, and three levels of confining pressure were used: 49, 98, and 196 kN/m^2^. Drainage was from both end faces at a strain rate of 0.1%, and the pore water pressure was measured during shearing to ensure that no excess pore water pressure would be generated. All the UC tests, UC tests under water immersion, and CD tests were conducted on the sand-gel specimens for 1 to 300 days of material aging.

The hydrogel specimens were mechanically tested using the UC tests. The material age of the specimens ranged from 1 to 300 days. Volumetric shrinkage tests (VS tests) were performed on the hydrogel specimens before the UC tests. The diameter and height of the hydrogel specimens were measured with a digital caliper. The hydrogel specimens cured in glass capillaries of ϕ0.15 mm were observed until they had reached volumetric shrinkage. The hydrogel specimens cured in glass capillaries of ϕ2 mm were subjected to SAXS tests in order to analyze the microscopic molecular structure of the specimens.

Table 1 summarizes the tests conducted in this study along with the specimens and silica concentrations in the chemical solutions corresponding to each test.

### 2.3. Small-Angle X-ray Scattering Tests (SAXS Tests)

The small-angle X-ray scattering test (SAXS test) is a test in which a targeted object is irradiated with X-rays as incident light, and then scattered X-rays with a scattering angle of a few degrees or less are observed [16]. As SAXS tests can determine structural parameters in the range of 1 nm to 1 μm in size for targeted objects, the authors thought that it might be possible to analyze the chemical changes in the microscopic molecular structure of the hydrogel.

**Figure 2:**
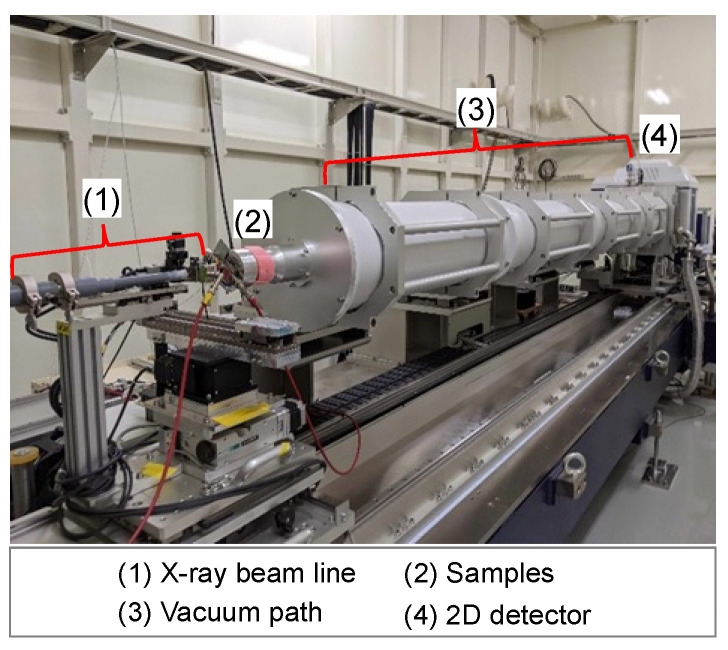
Appearance of beamline BL8S3.

**Figure 3:**
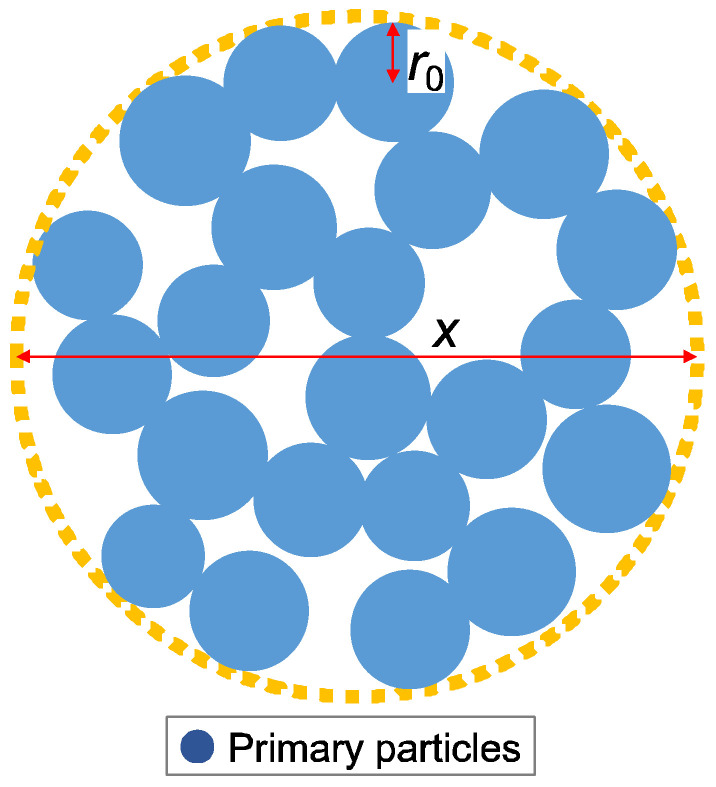
Schematic diagram of analytical model for aggregate structure.

The procedure for the SAXS tests is as follows:

(1) A quartz glass tube of ϕ2 mm for X-ray diffraction is filled with the chemical solution and sealed at the end.
(2) After gelation of the chemical solution, the tubes are cured at different curing temperatures of 10, 20, 45, and 60 °C.
(3) The SAXS tests are conducted on the hydrogel specimens at each material age, namely, 1, 7, 14, 21, and 28 days. The SAXS test apparatus used for the tests was the beamline BL8S3 (see Figure 2) operated at the Aichi Synchrotron Radiation Center, Japan.
(4) The measurement data obtained through the SAXS tests are fitted using the aggregate analysis model shown in Figure 3, and lower limit censoring length (assumed to be distributed in the analysis) r_0_, mass fractal dimension D, and censored length (correlation length) ξ are calculated.
(5) Simulated visualization is performed using ATSAS [17] for preprocessing, while ab initio modeling, using SasView [18], is employed to analyze the hierarchical structure.

## 3. Results and Discussion

### 3.1. UC Tests for Sand-Gel Specimens

**Figure 4:**
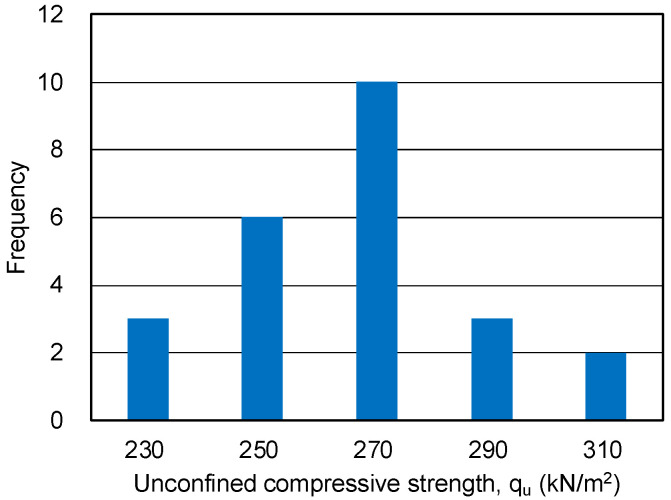
Histogram of unconfined compressive strength of sand-gel specimens.

The maximum and minimum unconfined compressive strengths of the 24 sand-gel specimens were 318.4 and 225.2 kN/m^2^, respectively, with an average value of 266.2 kN/m^2^. The standard deviation, which indicates variability, was 22.3 kN/m^2^, and the coefficient of variation was calculated as 8.4%. A histogram of the unconfined compressive strength of the sand-gel specimens is shown in Figure 4.

Table 2 shows the specifications of the sand-gel specimens. The coefficients of variation in wet density and water content are 0.5 and 2.2%, respectively. Hydrogel existed in the pores of the sand-gel specimens. As hydrogel comprises mostly water, water and silica were combined and evaluated as the water content for the calculation of the water content and degree of saturation.

### 3.2. UC Tests under Water Immersion for Sand-Gel Specimens

**Figure 5:**
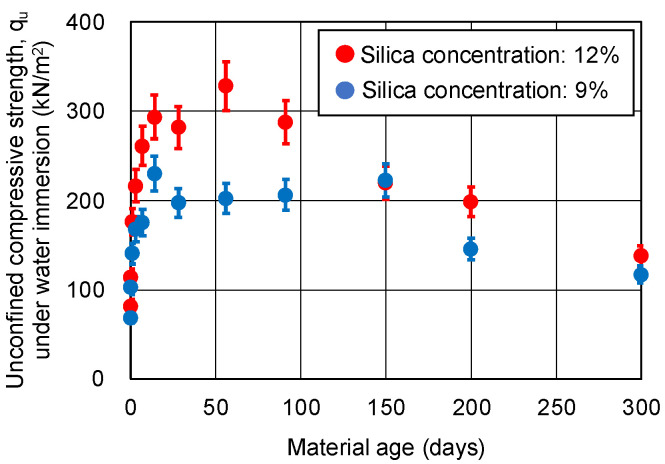
Unconfined compressive strength of sand-gel specimens under water immersion.

The results of the UC tests under water immersion are shown in Figure 5. The data in this figure are the averages of two specimens. Assuming that the coefficient of variation is the same for all of the sand-gel specimens, a coefficient of variation of 8.4% is shown as the error range. The unconfined compressive strength under water immersion tends to peak between 56 and 150 days of material aging and then decreases beyond the error range.

### 3.3. UC Tests for Hydrogel Specimens

**Figure 6:**
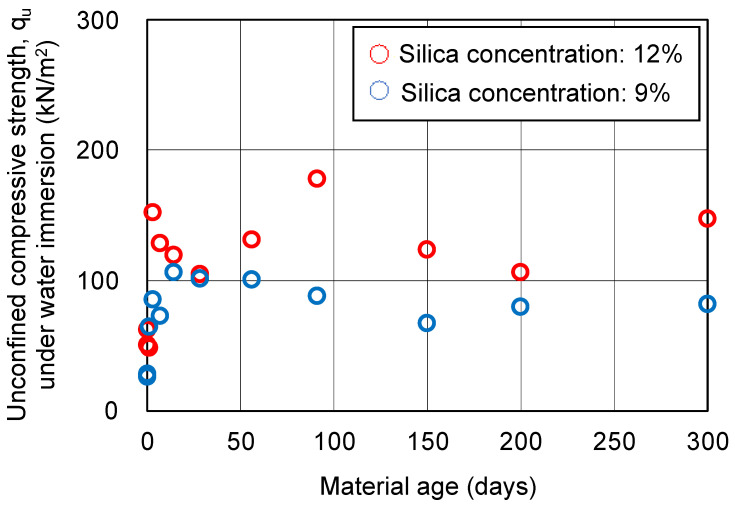
Unconfined compressive strength of hydrogel specimens under water immersion.

**Figure 7:**
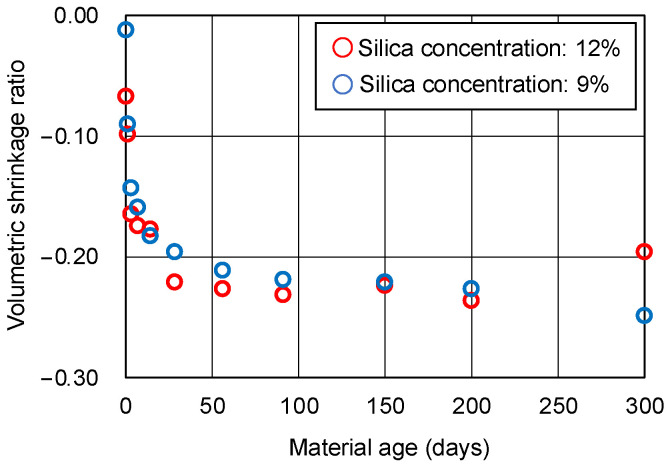
Volumetric shrinkage of hydrogel specimens.

The results of the UC tests on the hydrogel specimens are shown in Figure 6. The data in this figure are the averages of two specimens. Before the UC tests, the hydrogel specimens were measured with a digital caliper to calculate the volumetric shrinkage. Cracking of the hydrogel specimens during curing is inevitable for hydrogel of a certain volume. Therefore, changes in the unconfined compressive strength over time are considered as reference values for which no trend can be found. The volumetric shrinkage of the hydrogel specimens is shown in Figure 7. The results may also be inaccurate due to the presence of cracks. However, after 50 days of material aging for the hydrogel specimens, the shrinkage is seen to settle down.

### 3.4. CD Tests for Sand-Gel Specimens

**Figure 8:**
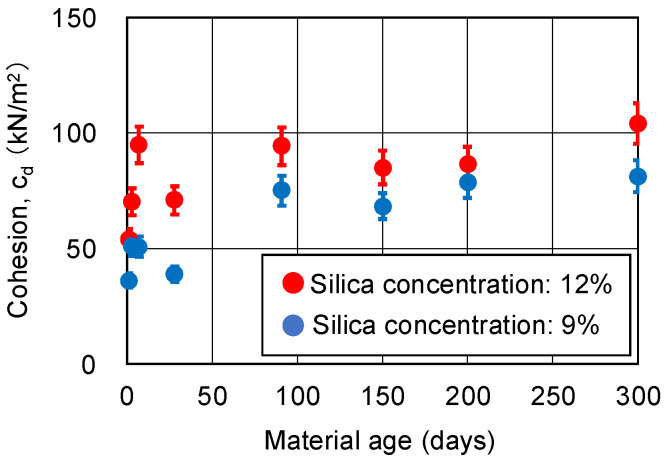
Cohesion of sand-gel specimens.

**Figure 9:**
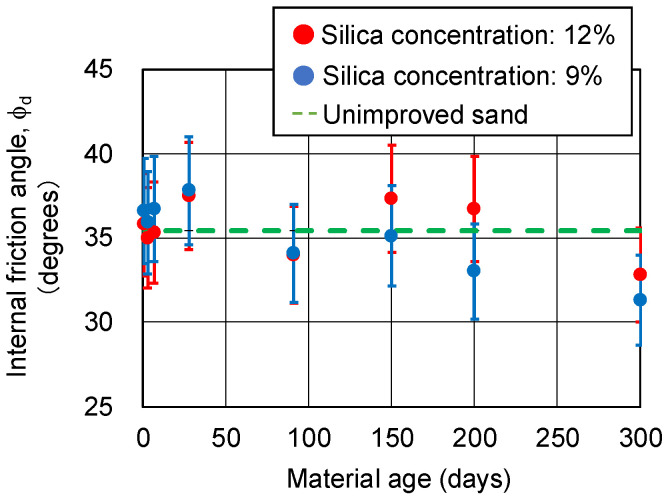
Internal friction angle of sand-gel specimens.

Figure 8 and Figure 9 show the changes over time in cohesion cd and internal friction angle ϕ_d_, respectively, obtained from the CD tests. The CD tests were conducted on three specimens under the same conditions, which resulted in variations among the specimens. Therefore, the coefficient of variation is expected to be larger than the unconfined compressive strength. However, since there are no data on the variation for these CD tests, a coefficient of variation of 8.4%, which is the same as the unconfined compressive strength, is assumed here as the error range. The cohesion cd of the sand-gel specimens (sandy soil improved via chemical injection) did not decrease even after 300 days. Internal friction angle ϕ_d_ was distributed around 35.4 degrees; this is equivalent to the internal friction angle of the unimproved sand. The variation is thought to be within the error range.

#### 3.4.1. Relationship between Strength Components and Axial Strain

Schmertmann [19,20] found that a failure envelope can be approximated linearly to Mohr’s effective stress circle obtained when the magnitude of axial strain is taken as a parameter. The cohesion and internal friction angle at each axial strain were obtained from the failure envelopes corresponding to each axial strain [19,20].

**Figure 10:**
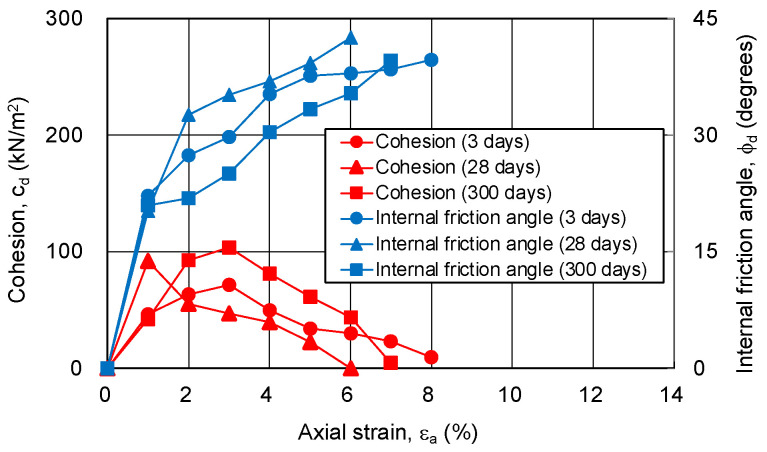
Cohesion and internal friction angle for changing axial strain of sand-gel specimens (silica concentration: 9%).

**Figure 11:**
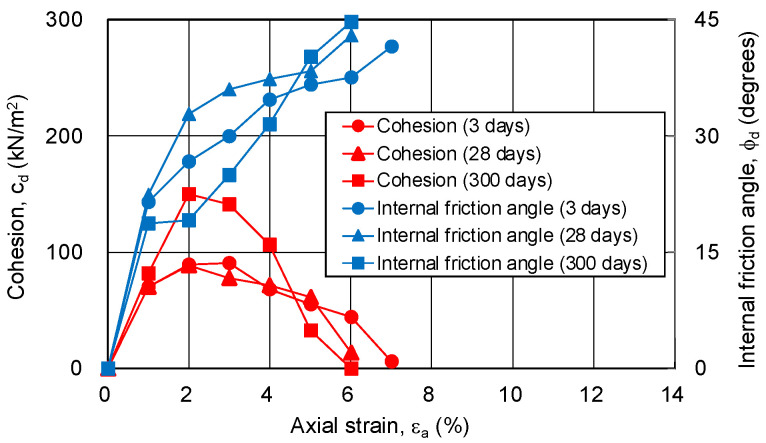
Cohesion and internal friction angle for changing axial strain of sand-gel specimens (silica concentration: 12%).

In this study, the cohesion and internal friction angle, corresponding to the magnitude of axial strain, were obtained using the procedure proposed by Schmertmann [19,20]. Firstly, a Mohr’s stress circle was plotted for each axial strain based on the principal stress difference and confining pressure. Next, the cohesion and internal friction angle, corresponding to the axial strain, were obtained through drawing a fracture collapse line on the stress circle. The results are shown in Figure 10 and Figure 11. The relationship among the cohesion, internal friction angle, and axial strain is the same regardless of the silica concentration and material age. The cohesion shows a maximum value at low strain (around 2%), and then decreases to zero. The internal friction angle increases with strain and finally exceeds 40 degrees. This indicates that, as the shear deformation progresses, the shear strength component resisting the shear deformation changes.

**Figure 12:**
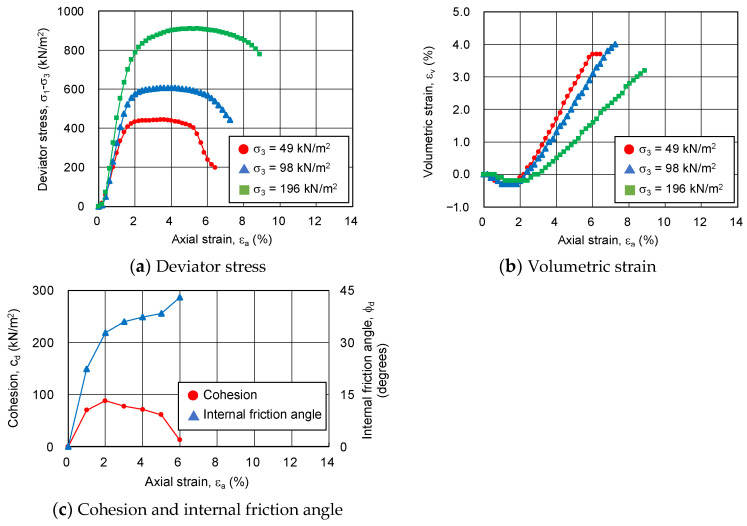
Main stress difference, volumetric strain, cohesion, and internal friction angle for changing axial strain of sand-gel specimens.

In order to clarify the above-mentioned process of shear deformation, changes in deviator stress, volumetric strain, cohesion, and internal friction angle with respect to axial strain are compared. Figure 12 shows the deviator stress, volumetric strain, cohesion, and internal friction angle as functions of axial strain at 12% silica concentration and 28 days of material age. Cohesion is at its maximum at an axial strain of around 2%, where the compressive strain is at its maximum. At an axial strain of around 5%, where the deviator stress shows a decreasing trend from the maximum value, volume expansion continues due to positive dilatancy, but cohesion remains at 61 kN/m^2^, indicating that the hydrogel has not been destroyed. However, as the axial strain continues to increase over 5%, cohesion becomes zero, i.e., the hydrogel on the failure surface has been destroyed. On the other hand, the internal friction angle continues to increase above the internal friction angle of the unimproved sandy soil until the hydrogel fails. The internal friction angle alone is responsible for the shear resistance in terms of residual strength.

#### 3.4.2. Relationship between Strength Components and Hvorslev Failure Criteria

Hvorslev failure criteria [21,22,23] were applied to quantitatively evaluate the relationship between the volume change and strength components during the shear process. The more densely packed the sandy soil particles are, the larger the contact area between them. As a result, the cohesive resistance of the contact surface tends to increase and the value of the coefficient of friction also tends to increase. Hvorslev considered that the true cohesion, the strength component of sandy soil, is a function of the pore ratio at the failure surface of the sandy soil and is independent of the stress history and the structure of the sandy soil [21,22,23].

In this study, the idea that cohesion is a function of the pore ratio was incorporated into the Hvorslev failure criteria. In other words, the authors considered that the changes in volume due to the axial strain of the sand-gel specimens correspond to the changes in pore ratio, and evaluated the correspondence between the changes in pore ratio and the changes in cohesion of the sand-gel specimens.

The evaluation procedure is as follows:

(1) The wet density at each axial strain is calculated from the volume change (amount of water absorbed or drained) at each axial strain.
(2) The pore ratio at each axial strain is calculated from the sandy soil particle density, saturation, and wet density.
(3) The pore ratio is related to the change in cohesion.

The following assumptions were made in the evaluation procedure:

(1) Consolidation and shearing cause changes in volume, and the amount of drainage or water supply at these times correspond to changes in the volume and mass of the sand-gel specimens (density of 1.0 g/cm^3^ as water).
(2) The saturation of the specimens does not change from before shearing up to the end of the CD test.

**Figure 13:**
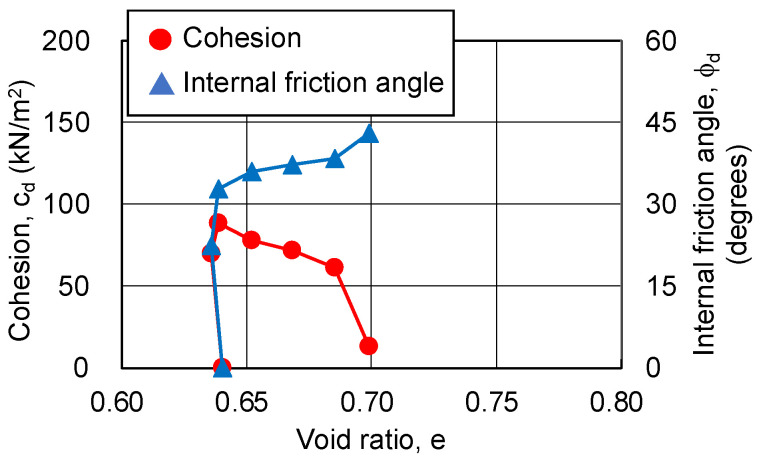
Cohesion and internal friction angle for changing pore ratio on sand-gel specimens with silica concentration of 12%, material age of 28 days, and σ_3_ = 49 kN/m^2^.

**Figure 14:**
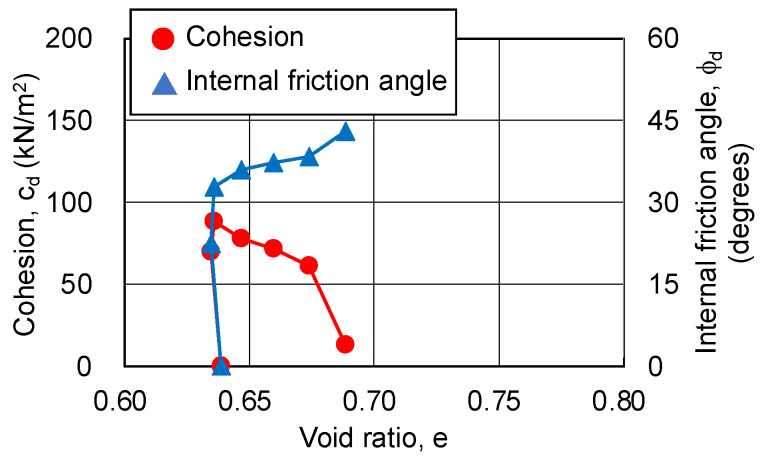
Cohesion and internal friction angle for changing pore ratio on sand-gel specimens with silica concentration of 12%, material age of 28 days, and σ_3_ = 98 kN/m^2^.

**Figure 15:**
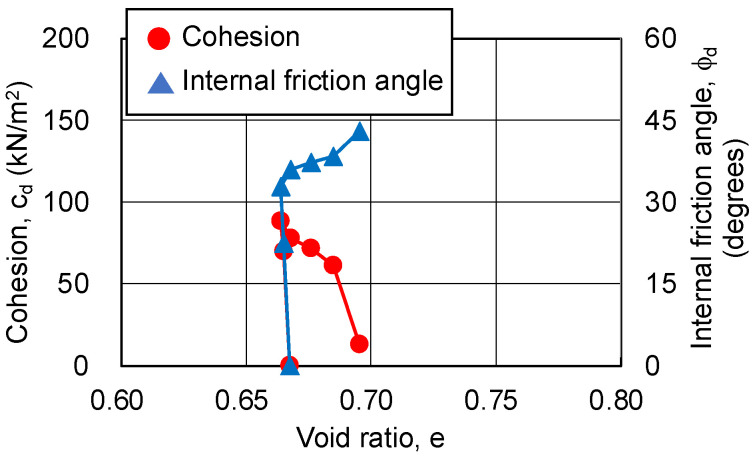
Cohesion and internal friction angle for changing pore ratio on sand-gel specimens with silica concentration of 12%, material age of 28 days, and σ_3_ = 196 kN/m^2^.

As the changing trends in the pore ratio and cohesion are similar, regardless of the silica concentration and material age of the sand-gel specimens, Figure 13, Figure 14 and Figure 15 show the results for a silica concentration of 12% and a material age of 28 days for the sand-gel specimens as an example. From these figures, cohesion is found to show its maximum value at the minimum pore ratio (maximum compression). Since consolidation has been completed, the decrease in pore ratio until the maximum compression has been reached is as small as 0.003. After the pore ratio shows a minimum value and then begins to increase (volume expansion), the cohesion decreases approximately proportionally. At this time, the pore ratio changes from 0.03 to 0.06, a change of more than 10 times compared to the time when compression is at its maximum. As the confining pressure increases, the range of changes in the pore ratio becomes smaller. The internal friction angle increases rapidly to about 30 degrees during compression (during drainage) and continues to increase slowly during volume expansion (during water absorption), finally exceeding 40 degrees.

### 3.5. Volumetric Shrinkage Tests for Hydrogel Specimens

**Figure 16:**
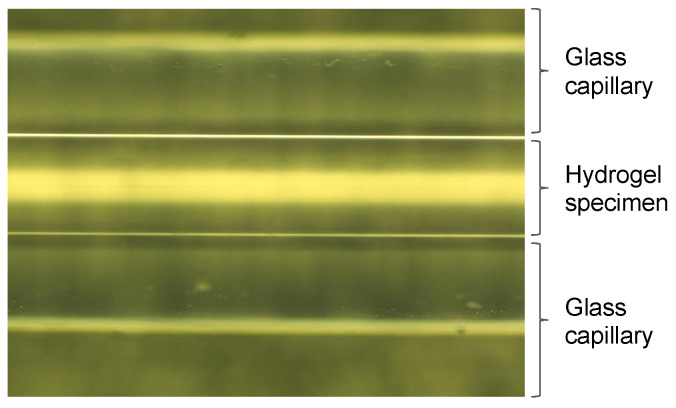
Glass capillary at curing temperature of 60 °C and material age of 28 days.

No volumetric shrinkage was observed for the hydrogel specimens in the glass capillary of ϕ0.15 mm simulating the pore space of coarse sandy soil, regardless of the conditions of the curing temperature (see Figure 16).

### 3.6. SAXS Tests for Hydrogel Specimens

**Figure 17:**
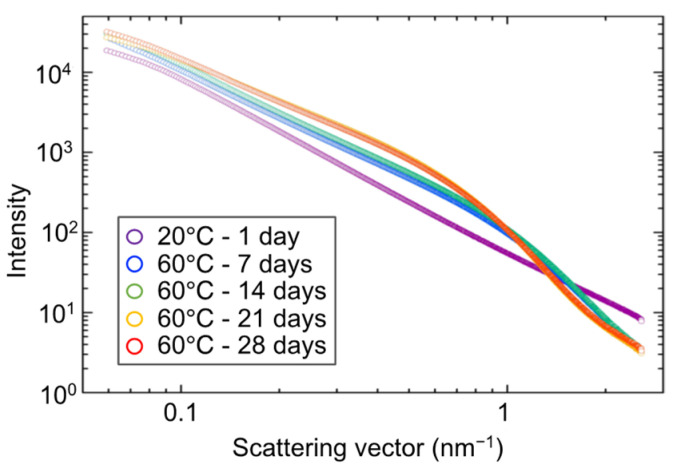
Scattering curves of hydrogel specimens.

The results of the small-angle X-ray scattering tests are organized as scattering curves. An example of these scattering curves is shown in Figure 17. The slope of the scattering vector below 1 nm^−1^ is determined using the size information (correlation length) of the measured structure. The slope of the area around the scattering vector, 0.1 to 1 nm^−1^, is determined using the mass fractal dimension of the measured structure. The mass fractal dimension is a parameter that indicates the degree to which a given volume is filled with particles.

**Figure 18:**
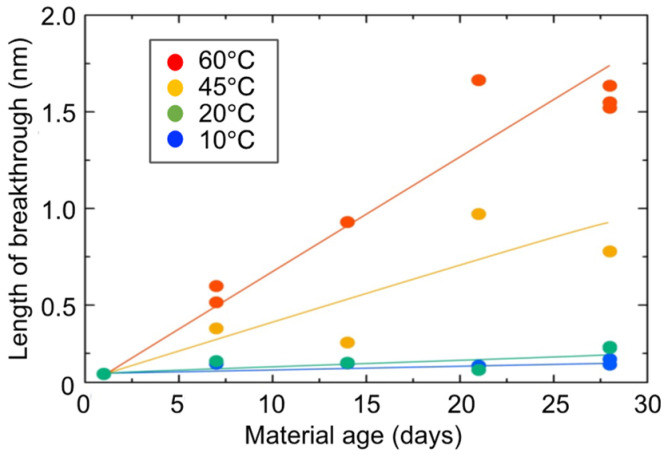
Changes over time in primary diameter (size) of hydrogel specimens.

Figure 18 shows the changes over time of the lower limit of the length of breakthrough r_0_. r_0_ corresponds to the primary diameter of a particle, which makes up the skeleton of each hydrogel specimen. At the curing temperature of 20 °C, r_0_ increases slowly, but at the curing temperature of 60 °C, r_0_ increases more rapidly. This suggests that the primary diameter (size) of a particle increases monotonically with time.

**Figure 19:**
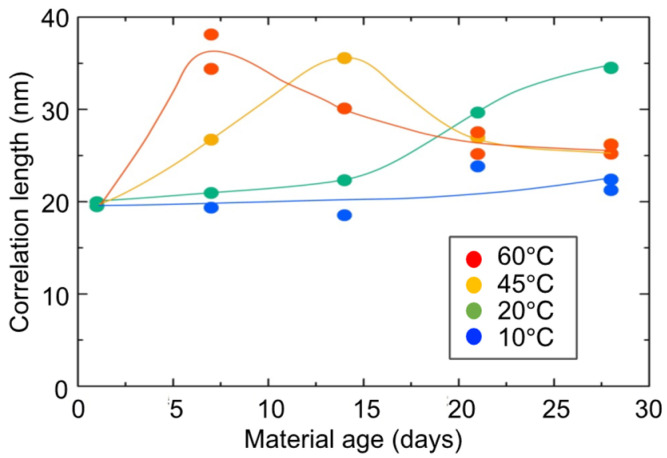
Changes over time in correlation length of hydrogel specimens.

Figure 19 shows the changes over time of the correlation length. The correlation length is a parameter that indicates the minimum size of the aggregate structure that behaves as a gel (or mesh structural size of the gel). Curing at higher temperatures corresponds to accelerated curing. Comparing the peaks of the correlation lengths, the acceleration is about four times greater at a curing temperature of 60 °C than at a curing temperature of 20 °C. The case of the 60 °C curing temperature shows that the correlation length increases once, reaches a maximum value, and then decreases. This means that the micro-aggregate structure (micro-mesh structure of the gel) that constitutes the gel has shrunk after growing once.

**Figure 20:**
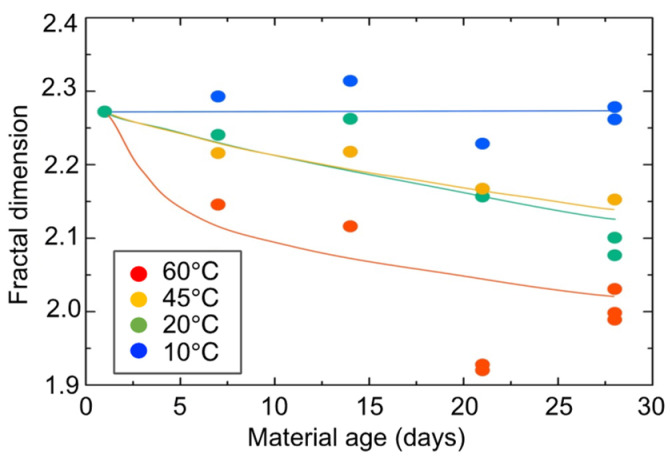
Changes over time in fractal dimension of hydrogel specimens.

Figure 20 shows the changes over time of the fractal dimension. The fractal dimension indicates the degree to which the aggregate is “packed” with the primary diameter (size) of particles. When the aggregate structure (mesh structure of the gel) is dense, the value approaches three, and when the structure is sparse, the value approaches one. The fractal dimension is seen to have decreased monotonically over time. The fractal dimension decreased rapidly during accelerated curing. This means that the primary diameter (size) of the particles became less clogged within the size range of the correlation length. Alternatively, the primary diameter (size) of the particles themselves may have become sparse.

**Figure 21:**
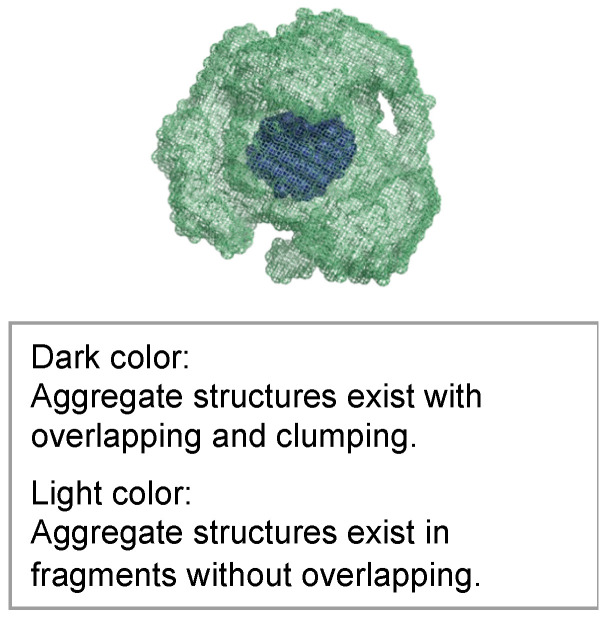
Molecular structure model of hydrogel specimens cured at 60 °C for 1 day.

**Figure 22:**
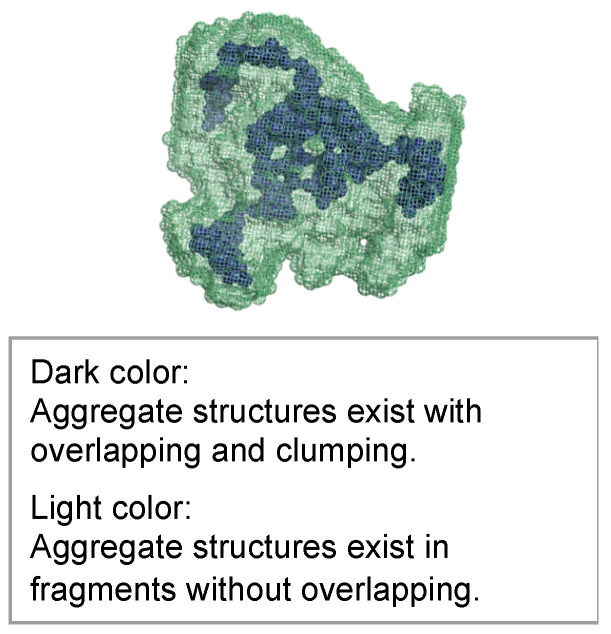
Molecular structure model of hydrogel specimens cured at 60 °C for 7 days.

**Figure 23:**
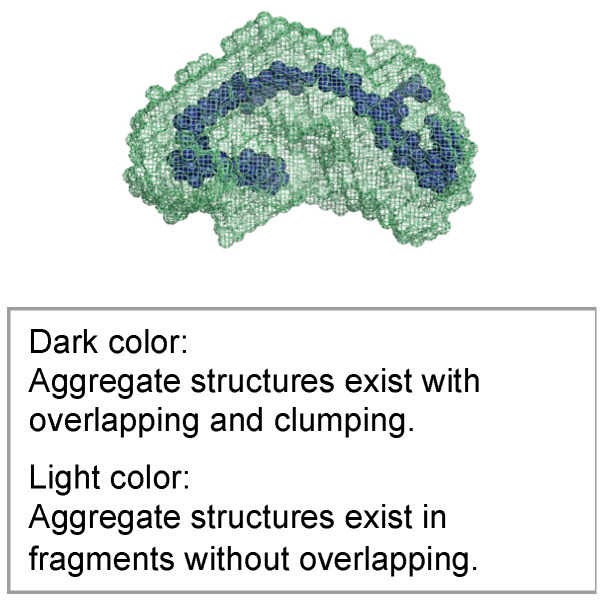
Molecular structure model of hydrogel specimens cured at 60 °C for 28 days.

The molecular structures obtained using ab initio modeling are shown in Figure 21, Figure 22 and Figure 23. This ab initio modeling is a method to analyze the structure of the scattering profile in Figure 16 via computer simulation using SasView [18]. The changes in the aggregate structure (mesh structure of the gel) of the hydrogel specimens over time can be summarized as follows. Assuming a curing temperature of 20 °C as the standard, the aggregate structure (mesh structure of the gel), expressed by the correlation length, grows from 20 to 35 nm from the material age of 14 days. The peak growth of the aggregate structure (mesh structure of the gel) corresponds to the material age of 28 days at a curing temperature of 20 °C and the material age of 7 days at a curing temperature of 60 °C. This is the result of ab initio modeling. As shown in the ab initio modeling results (see Figure 21 and Figure 22), the microstructure grew significantly and the correlation length increased, but the fractal dimension of the structure decreased due to the growth of side chains. As time passed, the size of the aggregate structure (mesh structure of the gel) decreased to about 25 nm and, at the timing when the aggregate structure (mesh structure of the gel) began to shrink (after a material age of 7 days under curing at 60 °C), the primary diameter (size) of the particles grew to about 0.5 nm and continued to increase to 1.5 nm. The degree of particle packing (fractal dimension) continued to decrease. At that time, as shown in Figure 23, a chain-like structure grew, and it is thought that the aggregate structure (mesh structure of the gel) was formed with this as the building block, resulting in a structure with a low fractal dimension. Since the primary diameter (size) of the particles continued to grow, it is presumed that the chains themselves, which constitute the mesh, became thicker. As the aggregate structure (mesh structure of the gel) became smaller, it was predicted that cracks (microcracks) would occur due to attractive forces between the structures.

### 3.7. Strength Assessment

The strength of the sandy soil improved via chemical injection is manifested by an increase in cohesion and the internal friction angle. These strength components change during the process of shearing. In the initial stage of shearing, the volume of the sandy soil improved via chemical injection decreases, and the hydrogel inside the improved soil is compressed to drain out the water contained in it. Correspondingly, cohesion and the internal friction angle increase. When the decrease in volume reaches its peak, cohesion reaches its maximum value. This is thought to be an effect of the hydrogel being compressed and drained. The volume of the sandy soil improved via chemical injection begins to increase. The sandy soil particles become untangled and the pore ratio increases. Cohesion decreases in proportion to the pore ratio, but the internal friction angle continues to increase. Even when the maximum axial differential stress is reached at the failure strain, cohesion remains, suggesting that the tensile failure of the hydrogel has not yet occurred. As the axial strain increases, the hydrogel at the failure surface eventually fails in tension and cohesion becomes zero. The internal friction angle continues to increase until this point, exceeding 40 degrees. This angle is much higher than that of the sandy soil before chemical injection; this is thought to be an effect of the fixation of the sandy soil particles caused by the solidification of the chemical solution. The fixation of the sandy soil particles was also pointed out by Mori and Tamura [7,8] and Mori et al. [9,10,11].

The above discussion suggests that the strength development of sandy soil improved via chemical injection firstly stems from the addition of and increase in cohesion, which is thought to have the effect of sandy soil particle fixation. The residual strength also showed that the fixation effect of the sandy soil particles continued, and that the adhesion between the sandy soil particles and the hydrogel was not broken off. It is presumed that water drainage and water supply take place inside the hydrogel of the sandy soil improved via chemical injection. Therefore, the failure of the sandy soil improved via chemical injection is believed to be the failure of the aggregate structure of the inside of the hydrogel itself, not interface failure.

The mechanism of the strength development of the sandy soil improved via chemical injection is similar to that of the specimen with the material age of 300 days, and the cohesion of the sandy soil improved via chemical injection is maintained even after 300 days. The fixation effect of the sandy soil particles is also demonstrated without degradation. Therefore, it is presumed that no detachment occurs at the interface between the hydrogel and the sandy soil particles.

Traditionally, the long-term decrease in unconfined compressive strength has been explained by the loss of adhesion between the hydrogel and the sandy soil particles due to the volumetric shrinkage of the hydrogel. However, the experimental results in this study suggest that neither the volumetric shrinkage of the hydrogel nor the detachment of the sandy soil particles occurs in the pore spaces of the sandy soil particles. In the CD tests with confining pressure, the strength components of the sandy soil improved via chemical injection, namely, cohesion and the internal friction angle, do not decrease even after the material age of 300 days.

On the other hand, the analysis of the microscopic molecular structure of the hydrogel showed the phenomenon in which the aggregate structure in the order of 10 nm became smaller. This may indicate the formation of microcracks in the hydrogel due to tensile stress. The decrease in the unconfined compressive strength of the hydrogel is the decrease in the apparent strength due to the absence of confining pressure, and may be due to microcracks in the hydrogel. The CD tests are considered to have little effect on the strength because the microcracks in the hydrogel are sealed due to confining pressure. The skeleton of the hydrogel grows with time; this may also be a reason why the strength is not reduced. As the material age of the hydrogel advances, it tends to exhibit the behavior of a more brittle failure, although the strength itself does not decrease. This is considered to be due to the effect of microcracks in the hydrogel.

The volumetric shrinkage of the hydrogel is addressed in relation to the specific surface area. Let us assume that the adhesion and volumetric shrinkage forces of the hydrogel are constant and that the volumetric shrinkage force is proportional to the volume of the hydrogel. The volumetric shrinkage of the hydrogel is a matter of specific surface area per unit volume. Calculating specific surface area Sv from the average diameter of particle Dw, using Equation (1) [24,25], Table 3 is obtained.

(1) $$Sv=\frac{6}{Dw}$$

Table 3 assumes that the diameters of the glass capillaries and molds are the respective average diameters. The average particle sizes of the standard sand and the glass capillary differ by more than two orders of magnitude in specific surface area compared to the respective average diameters of the molds. Therefore, because no volumetric shrinkage of the hydrogel was observed in the glass capillaries, it can be inferred that no volumetric shrinkage of the hydrogel occurs in the pore spaces between the sandy soil particles.

## 4. Conclusions

The results of this study are summarized as follows:

(1) The strength development of the sandy soil improved via chemical injection can be explained by the increases in cohesion and the internal friction angle of the sandy soil particles fixed by the chemical solution.
(2) Under triaxial conditions with confining pressure, such as in CD tests, the strength component of the sandy soil improved via chemical injection shows no long-term loss of strength.
(3) No physically visible volumetric shrinkage of the hydrogel occurs in the pore spaces of the soil particles.
(4) The failure of the sandy soil improved via chemical injection is the failure of the aggregate structure of the hydrogel itself.
(5) The aggregate structure inside the hydrogel changes to a sparse state due to the shrinkage of the microscopic molecular structure of the hydrogel over time.

No evaluation of the sandy soil improved via chemical injection has been actively performed because of the difficulty of preparing three homogeneous specimens. However, CD tests should be used to evaluate the strength component regardless of whether the sand-gel specimens are drained or undrained. Future issues to be addressed include an evaluation of the variability of the CD tests. The long-term strength of sandy soil improved via chemical injection can be evaluated from a chemical point of view by analyzing the aggregate structure inside the hydrogel specimens using SAXS tests. These tests can evaluate the internal structure of the hydrogel as a cohesive mass about the size of an aggregate structure (mesh structure of the gel), instead of conventional chemical degradation, which is the cleavage of the molecular structure (dissolution of the silica content). This method is thought to be an effective alternative to the model that evaluates the visible volumetric shrinkage of the hydrogel as physical degradation.

## Figures and Tables

**Table 1:** List of tests.

Test	Specimen	SiO_2_
Unconfined compression test (UC test)	Sand-gel	12%
	Hydrogel	12%, 9%
Unconfined compression test under water immersion (UC test under water immersion)	Sand-gel	12%, 9%
Consolidation drainage triaxial compression tests (CD test)	Sand-gel	12%, 9%
Volumetric shrinkage test (VS test)	Hydrogel	12%, 9%, 7%
Small-angle X-ray scattering test (SAXS test)	Hydrogel	7%

**Table 2:** Specifications of sand-gel specimens.

Basic Physical Properties Index			
Wet density	ρ_t_	1.995	g/cm^3^
Dry density	ρ_d_	1.591	g/cm^3^
Natural water content	w_n_	25.4	%
Void ratio	e	0.659	
Degree of saturation	S_r_	101.7	%
Relative density	D_r_	58.0	%

**Table 3:** Average particle size and specific surface area.

Materials	Average Diameter, Dw (cm)	Specific Surface Area per Unit Volume, Sv (cm^2^/cm^3^)
Standard sand	0.0191	314.1
Glass capillary	0.0150	400.0
Plastic mold	5.0000	1.2

## Data Availability

The data presented in this study are openly available in article.
